# Monocyte anisocytosis increases during multisystem inflammatory syndrome in children with cardiovascular complications

**DOI:** 10.1186/s12879-022-07526-9

**Published:** 2022-06-20

**Authors:** Lael M. Yonker, Oluwakemi Badaki-Makun, Puneeta Arya, Brittany P. Boribong, Gabriela Moraru, Brittany Fenner, Jaimar Rincon, Alex Hopke, Brent Rogers, Jeremiah Hinson, Alessio Fasano, Lilly Lee, Sarah M. Kehoe, Shawn D. Larson, Hector Chavez, Scott Levin, Lyle L. Moldawer, Daniel Irimia

**Affiliations:** 1 Department of Pediatrics, Massachusetts General Hospital, 55 Fruit Street, Boston, MA 02114 USA; 2 Mucosal Immunology and Biology Research Center, Massachusetts General Hospital, 55 Fruit Street, Boston, MA 02114 USA; 3 Harvard Medical School, Boston, MA USA; 4 Department of Pediatrics, Johns Hopkins School of Medicine, Baltimore, MD USA; 5 Center for Data Science in Emergency Medicine, Johns Hopkins University, Baltimore, MD USA; 6 Jackson Memorial Hospital, Miami, FL USA; 7 Holtz Children’s Hospital, Miami, FL USA; 8 Department of Surgery, University of Florida, Gainesville, FL USA; 9 Danaher Diagnostics LLC, Boston, MA USA; 10 Department of Emergency Medicine, Johns Hopkins School of Medicine, Baltimore, MD USA; 11 Department of Surgery, Center for Engineering in Medicine, Massachusetts General Hospital, 114 16th Street, Boston, MA 02129 USA; 12 Shriners Burn Hospital, Boston, MA USA

**Keywords:** Pediatric COVID-19, Multisystem inflammatory syndrome in children, Monocyte distribution width

## Abstract

**Background:**

Multisystem inflammatory syndrome in children (MIS-C) is a life-threatening complication that can develop weeks to months after an initial SARS-CoV-2 infection. A complex, time-consuming laboratory evaluation is currently required to distinguish MIS-C from other illnesses. New assays are urgently needed early in the evaluation process to expedite MIS-C workup and initiate treatment when appropriate. This study aimed to measure the performance of a monocyte anisocytosis index, obtained on routine complete blood count (CBC), to rapidly identify subjects with MIS-C at risk for cardiac complications.

**Methods:**

We measured monocyte anisocytosis, quantified by monocyte distribution width (MDW), in blood samples collected from children who sought medical care in a single medical center from April 2020 to October 2020 (discovery cohort). After identifying an effective MDW threshold associated with MIS-C, we tested the utility of MDW as a tier 1 assay for MIS-C at multiple institutions from October 2020 to October 2021 (validation cohort). The main outcome was the early screening of MIS-C, with a focus on children with MIS-C who displayed cardiac complications. The screening accuracy of MDW was compared to tier 1 routine laboratory tests recommended for evaluating a child for MIS-C.

**Results:**

We enrolled 765 children and collected 846 blood samples for analysis. In the discovery cohort, monocyte anisocytosis, quantified as an MDW threshold of 24.0, had 100% sensitivity (95% CI 78–100%) and 80% specificity (95% CI 69–88%) for identifying MIS-C. In the validation cohort, an initial MDW greater than 24.0 maintained a 100% sensitivity (95% CI 80–100%) and monocyte anisocytosis displayed a diagnostic accuracy greater that other clinically available hematologic parameters. Monocyte anisocytosis decreased with disease resolution to values equivalent to those of healthy controls.

**Conclusions:**

Monocyte anisocytosis detected by CBC early in the clinical workup improves the identification of children with MIS-C with cardiac complications, thereby creating opportunities for improving current practice guidelines.

## Background

Multisystem inflammatory syndrome in children (MIS-C) is a life-threatening complication of COVID-19 that develops in children, weeks to months after the initial SARS-CoV-2 infection, which may have been mild or asymptomatic [1]. With the emergence of novel SARS-CoV-2 variants of concern, waning mRNA vaccine and natural immunity, variable masking policies, and vaccine hesitancy, the cases of children with severe, life-threatening MIS-C will remain a medical concern for the foreseeable future. Although advances have been made to define the underlying pathology of MIS-C [2–4], the process required to distinguish MIS-C from other infectious illnesses in the clinic remains complex and time-consuming [5]. Clinicians are left to rely on clinical phenotype and extensive testing to identify children with MIS-C and determine whether treatment is necessary [6, 7].

While MIS-C is associated with diffuse immune activation and dysregulation [9], evidence suggests monocyte activation [2, 10, 11], persistence of patrolling monocytes [11] and a subsequent cytokine storm [2] are a vital component of the dysfunctional hyperinflammatory responses during MIS-C. Recent findings support a role for SARS-CoV-2 antigenemia in triggering a superantigen-like hyperinflammatory response [4, 12, 13], and expanding humoral and cellular responses [14] that activate monocytes [2, 15]. However, despite our advances in understanding the pathology driving MIS-C, clinically available laboratory tests have limited ability to capture this immune cell dysfunction and hyperactivation.

In this study, we assessed whether hematologic parameters could aid in evaluating children with persistent fever and offer early guidance towards the early identification of children with cardiovascular MIS-C. Because monocyte activation plays a key role in the hyperinflammatory responses of MIS-C and monocyte anisocytosis, which can be quantified by monocyte distribution width (MDW), has been shown to be a useful biomarker for sepsis and organ dysfunction in children [16, 17] and adults [18–20], and has now achieved Food and Drug Administration (FDA) clearance as a biomarker for sepsis in adults, we evaluated whether monocyte anisocytosis could aid in the identification of children with MIS-C. Monocyte anisocytosis can be measured with a hematology analyzer as part of a routine CBC, improving its utility and offering early guidance in the evaluation of a child for MIS-C.

## Methods

**Fig. 1:**
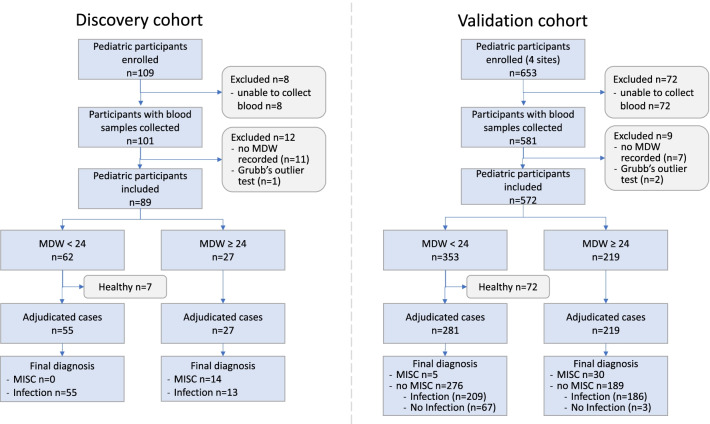
Overview of participants enrolled in discovery and validation cohorts to analyze MDW as a hematologic marker of MIS-C. The final analysis compared the MDW values in blood samples from MIS-C vs. infection/inflammation subjects. Healthy subjects were excluded from the final analysis and analyzed separately to establish normative MDW values in children

Pediatric patients 21 years old or younger who sought medical care from April to October of 2020 at Massachusetts General Hospital were prospectively enrolled in the discovery cohort to test the hypothesis that MDW was associated with MIS-C and establish a cut-off threshold for MIS-C screening (MGB IRB #2020P000955) [21]. The validation cohort prospectively tested this MDW threshold for MIS-C by enrolling children presenting for medical care during the COVID-19 pandemic from October 2020 to October 2021 at participating institutions (Massachusetts General Hospital, Boston, MA; Johns Hopkins Children’s Center, Baltimore, MD; Shands-University of Florida Health Science Center, Gainesville, FL; Jackson Memorial Hospital, Miami, FL) (MGB IRB #2020P002961), Fig. 1. Informed consent and assent when appropriate, was obtained from all participants and/or parents/legal guardians. For both the discovery and validation cohorts, any pediatric patient (≤ 21 years of age) seeking medical care during the defined time periods and consented/assented to provide a blood sample, was eligible for participation. All procedures and experiments were performed in accordance with IRB guidelines.

A blood sample was collected via venipuncture in a phlebotomy tube containing di-potassium ethylenediaminetetraacetic acid (K2 EDTA) anticoagulant and analyzed on the DxH900 Hematology Analyzer (Beckman Coulter, Brea, CA) using experimental protocols (MGB Institutional Biosafety Committee approval #2020B000061). Monocytes were identified by light scatter, volume, and conductivity. Monocyte volume of individual cells was measured by impedance using the Coulter principle. [22] MDW was automatically calculated as the standard deviation of monocyte volume divided by the mean monocyte volume and multiplied by 100 to express data as a percentage [19]. Metadata were extracted from medical records and managed using REDCap electronic data capture tools hosted at Massachusetts General Hospital. [23] Repeat blood collections were obtained in hospitalized MIS-C patients when possible. Of note, some patients were either unable to provide a blood sample or MDW was not calculated (Fig. 1).

Children were categorized into the following groups: (1) “MIS-C”: per CDC criteria [24] (2) “Infectious”: acute COVID-19 or other infections, (3) “Non-infectious illness”: children presenting for urgent medical care without fever or other signs of infection, (4) “Healthy controls”: asymptomatic children presenting for routine medical care. If a clinical diagnosis of MIS-C was reported, two study staff members blinded to the MDW values independently reviewed the case to confirm MIS-C diagnosis. A pediatric cardiologist, also blinded to MDW values, adjudicated MIS-C cases and verified whether patients met criteria for cardiac involvement of MIS-C, using previously established criteria for ventricular dysfunction, coronary aneurysm, vasopressor support, myocarditis [25].

Statistical analysis was performed using Prism (GraphPad Software version 9.2) and SPSS Statistics (IBM) using one-way-ANOVA parametric test with Tukey’s posthoc test. Single outliers were identified by Grubb’s outlier test and removed from the analysis. An Area Under the Receiver Operator Curve (AUROC) was calculated for the ability of MDW to distinguish MIS-C from other infectious or inflammatory processes. We estimated the sample size required to estimate true prevalence with a specified level of confidence and precision, assuming a test with imperfect sensitivity and/or specificity using previously published methods [26]. Graphs were prepared using Prism 9.2.

## Results

We enrolled a total of 762 children ≤ 21 years of age in a multicenter observational study and collected 846 blood samples for analysis across two study cohorts. A discovery cohort (n = 109 children) helped determine the MDW threshold for identifying children with MIS-C among children presenting to the ED with persistent fever and other illnesses (Fig. 1). A test cohort (n = 653 children) assessed the utility of MDW as a tier 1 assay for evaluating children presenting to the ED with persistent fever and other illnesses. Across the two cohorts, the mean age of participants was 10 years (range 4 days–21 years), with a near equal number of males and females (51% male, 49% female). By race, participants were White (n = 399, 52%), Black (n = 172, 23%), and Asian (n = 27, 4%). One-third (n = 231) were Hispanic/Latino (Table 1). A total of 57 children with MIS-C were enrolled (n = 17 and 40 in the discovery and validation cohorts, respectively). Characteristics of the children with MIS-C are included in Table 2. Of the 57 MIS-C cases, 27 (47%) had documented COVID-19 prior to their ED visit and 41 (72%) had SARS-CoV-2 detected on pcr of the nasal swab on admission. Forty-six had a known close contact with COVID-19, and 55 (96%) displayed antibodies against SARS-CoV-2 (Table 2). A total of 535 children presented with various other causes of infection/inflammation (n = 79 and 456; discovery, validation), of whom 98 had COVID-19. A total of 83 children presented with non-infectious causes of illness (such as trauma, syncope, etc., n = 0 and 83; discovery, validation). A total of 87 children were considered healthy (n = 13 and 74; discovery, validation). Eighty children across both cohorts were excluded because they were unable to provide a blood sample, or an MDW was not reported.

**Table 1:** Participant demographics

	Discovery cohort (n = 109)	Validation cohort (n = 653)	Total enrolled (n = 762)
Age years, mean (min,max)	10 (1 month–21)	10 (4 days–21)	10 (4 days–21)
Sex, n (%)			
Male	63 (58)	327 (50)	390 (51)
Female	46 (42)	326 (50)	372 (49)
Race, n (%)			
White	38 (35)	361 (55)	399 (52)
Black	7 (6)	165 (25)	172 (23)
Asian	8 (7)	19 (3)	27 (4)
Ethnicity, n (%)			
Hispanic	58 (53)	173 (26)	231 (30)
Illness classification, n (%)			
MIS-C	17 (16)	40 (6)	57 (8)
Infectious	79 (72)	456 (70)	535 (70)
Non-infectious	0 (0)	83 (13)	83 (11)
Healthy controls	13 (9)	74 (11)	87 (11)

A total of 762 pediatric patients have been enrolled in this study: 109 children in the Discovery Cohort (April–October 2020) and 653 children in the Validation Cohort (October 2020–October 2021). Demographics and disease classification are listed

**Table 2:** MIS-C patient characteristics

*MIS-C characteristics (N = 57)*	
Age, mean (min–max)	9 (2mo–21 years)
Sex, n (%)	
Male	33 (58)
Female	24 (42)
Race, n (%)	
White	26 (46)
Black	19 (33)
Asian	3 (5)
Other	11 (19)
Ethnicity, n (%)	
Hispanic	36 (63)
*MIS-C criteria*	
Evidence of prior SARS-CoV-2 infection/exposure	
Prior ( +) SARS-CoV-2 PCR	27 (47)
Current ( +) SARS-CoV-2 PCR	41 (72)
Current ( +) SARS-CoV-2 antibody test	55 (96)
Close exposure to individual with COVID-19	46 (80)
Fever, n (%)	57 (100)
Organ involvement, n (%)	
Cardiac	40 (70)
Ventricular dysfunction	14 (35)
Coronary aneurysm	6 (15)
Vasopressor support	14 (35)
Myocarditis	20 (50)
Gastrointestinal	53 (93)
Respiratory	36 (63)
Neurologic	26 (46)
Dermatologic	23 (40)
Mucocutaneous	22 (39)
Musculoskeletal	15 (26)
Renal	12 (21)

57 children with MIS-C enrolled in study. Demographics and clinical/laboratory evidence supporting MIS-C diagnosis are described

### Monocyte anisocytosis increases during MIS-C

**Fig. 2:**
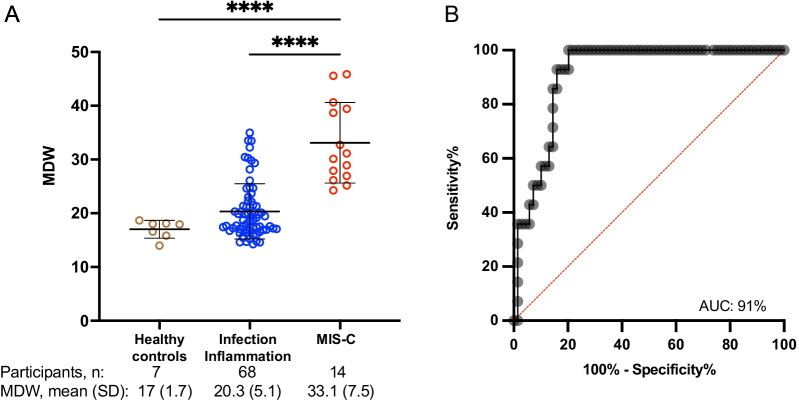
Monocyte anisocytosis is associated with MIS-C. **A** Discovery Cohort: Monocyte Distribution Width (MDW, a measure of monocyte anisocytosis) was quantified in blood samples from children with MIS-C, other infectious or inflammatory illnesses, and healthy controls. Analysis by ordinary one-way ANOVA. ****P < 0.0001. **B** Receiver operator curve (ROC) was used to assess the ability of MDW to serve as a tier 1 assay for distinguishing children with MIS-C from other children. An MDW threshold of 24 was established and tested in this discovery cohort for the ability to distinguish MIS-C from other illnesses. AUC = area under the curve (percentage). Single outlier identified by Grubb’s outlier test in the infection-inflammation group was removed for this analysis

In previous studies in adults, monocyte anisocytosis characterized by an MDW > 20 was associated with sepsis, and normative values were 20 or below [20]. While normative values have not yet been established for pediatrics, we analyzed MDW in healthy children in the discovery cohort. We determined that the healthy children had a mean MDW of 17.0 (min: 13.9, max 18.7, standard deviation [SD] 1.7), consistent with values seen in healthy adults. In contrast, children with MIS-C (n = 17 subjects enrolled, 14 with MDW values recorded) displayed a significant increase in monocyte anisocytosis with a mean peak MDW of 33.1 (min: 24.2, max 45.8, SD 7.5, Fig. 2A,  P < 0.0001).

Although children in the infections/inflammation group also displayed an increase in MDW as compared to healthy controls (Fig. 2A, P < 0.0001), MDW values in these other illnesses remained well below values seen in MIS-C (mean 20.3, min: 14.2, max 35.0, SD 5.1, Fig. 2A,  P < 0.001). Of note, peak MDW represented the first available blood collection in most children with MIS-C. Two children in the MIS-C group displayed a rising MDW up to 72 h after starting steroids and IVIG. Two children in the MIS-C group had the first blood sample collected 6 days post-treatment. For these children, we included the peak post-treatment values in the analysis.

Observing monocyte anisocytosis in MIS-C, we then sought to determine if MDW can distinguish MIS-C from other children presenting with illness. An area under the receiver-operator curve (AUROC) of 0.91 indicated that monocyte anisocytosis is highly associated with MIS-C (Fig. 2B). An MDW threshold of 24.0 provided the optimal screening cut-off to allow inclusive detection of all children with MIS-C while providing distinction from infectious controls (100% sensitivity, 81% specificity).

### Validation of the monocyte distribution width threshold in MIS-C

To validate the utility of an MDW threshold of 24.0 in distinguishing children with MIS-C, we calculated that we would need to enroll 31 MIS-C patients in our validation study (0.5 Type 1 error rate and at 80% power) based on the preliminary sensitivity and specificity determined in the discovery cohort. We estimated we would have to enroll 310 patients based on the entry criteria in endemic areas (~ 10% prevalence among ED patients enrolled). In practice, we evaluated MDW in 653 children participating in our multisite study from October 2020 to October 2021 (validation cohort, Table 1). In the validation cohort, we enrolled 40 children who met criteria for MIS-C, 35 of whom provided a blood sample from which an MDW was obtained. Additionally, we enrolled children presenting to the Emergency Department or hospital for medical care for infectious illness (n = 395) or non-infectious illness (n = 70). Blood samples were also collected from healthy controls (n = 72).

**Fig. 3:**
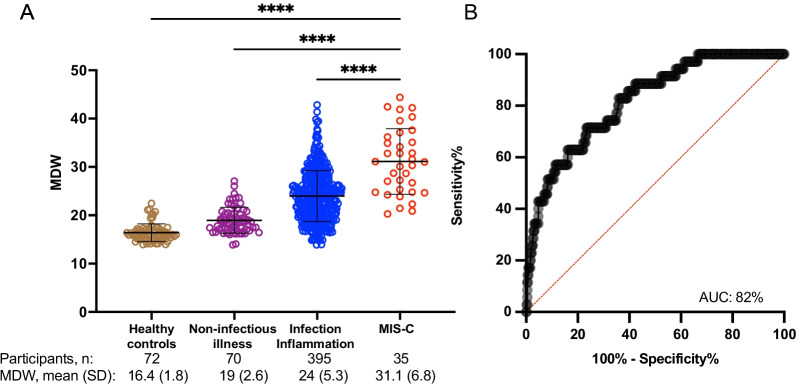
Testing MDW as a tier 1 assay of MIS-C. **A** Validation cohort: Blood samples were prospectively collected to assess an MDW threshold of 24 for identifying MIS-C. MDW was quantified in children with MIS-C, other infectious illnesses, non-infection illnesses, and healthy controls. Analysis by ordinary one-way ANOVA. ****P < 0.0001. **B** ROC in the validation cohort to assess the utility of MDW as a screening tool for MIS-C. AUC = area under the curve (percentage). Two outliers identified by Grubb’s outlier test in the healthy control and infection-inflammation groups were removed for analysis

In this larger validation cohort, monocyte anisocytosis was again identified as a robust tier 1 assay, with a higher mean in MIS-C (31.1, SD 6.8) compared to uninfected children (19.0, SD 2.6) and children with other infections (24.0, SD 5.3) (Fig. 3A, ANOVA P < 0.0001). The values of MDW in samples from healthy controls (16.5, SD 1.9) were comparable to those determined earlier in the discovery cohort (P = 0.49). Because MIS-C is defined by distinct hyperinflammatory responses, we sought to test the ability of MDW to identify MIS-C from a broad cohort of ill children presenting to the Emergency Department or upon admission to the hospital. Using the MDW cut-off threshold of 24.0 established in the discovery cohort, MDW was highly effective at distinguishing children with MIS-C from children with other illnesses (86% sensitivity, 60% specificity). An AUROC analysis revealed that MDW had high diagnostic accuracy (0.82) when comparing MIS-C (n = 35) to children presenting for medical care (infectious and non-infectious, n = 465) (Fig. 3B).

### Monocyte anisocytosis aids in identifying MIS-C with cardiac complications

**Fig. 4:**
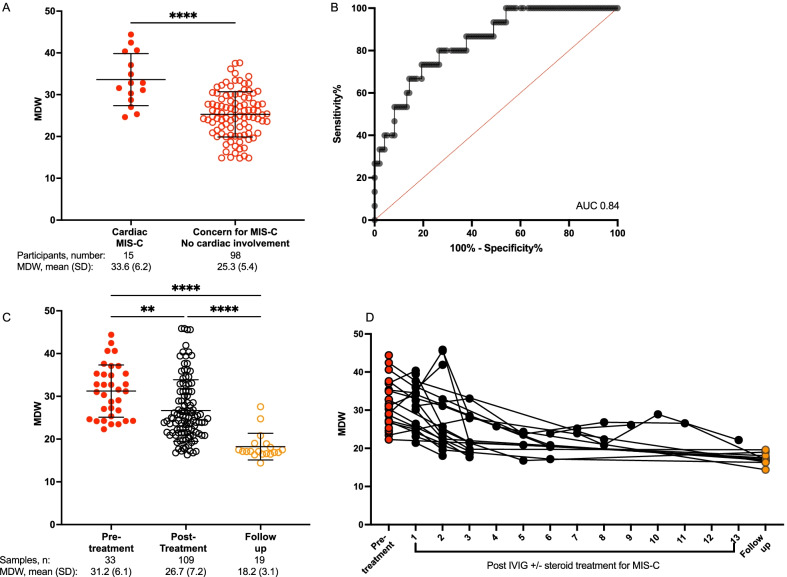
MDW depends on MIS-C severity and changes through the course of MIS-C diagnosis, treatment, and recovery. **A** Higher MDW values in MIS-C patients who manifested cardiac complications (Cardiac MIS-C) compared to children with MIS-C without cardiac involvement or presenting with symptoms concerning MIS-C (fever plus recent/current positive SARS-CoV2 PCR or SARS-CoV2 antibodies positive). **B** ROC in the validation cohort to assess the utility of MDW as a screening tool for cardiac involvement of MIS-C. AUC = area under the curve (fraction). **C** Blood from children with MIS-C was collected at multiple time points. MDW was plotted by time of collection: at admission, during hospital course, and at discharge or follow-up. Analysis by one way ANOVA. **P < 0.01, ****P < 0.0001. **D** MDW values from individual patients with MIS-C are plotted over the course of their illness. Black lines connect individual patients with MIS-C. Not all patients provided blood samples at each time point

Cardiovascular involvement of MIS-C has the greatest life-threatening potential and must be identified urgently to initiate treatments. As not all children with MIS-C will develop cardiac complications, we sought to ascertain whether MDW could aid in identifying MIS-C with cardiac complications specifically focusing on the development of myocarditis, ventricular failure, arrhythmias, coronary aneurysms, and/or cardiogenic shock. Myocarditis was defined as elevated troponin levels above the upper limit of laboratory normative values; ventricular failure was defined as ejection fraction < 55%; cardiac dysrhythmias and arrythmias were identified on electrocardiogram, coronary aneurysms were visualized by echocardiogram with a coronary artery z-score ≥ 2.5; and cardiogenic shock was identified by receipt of vasopressor or vasoactive support. [25] Cardiac involvement was confirmed by a pediatric cardiologist. We compared the cardiac MIS-C group to children presenting with symptoms concerning MIS-C but without the development of cardiac abnormalities. This non-cardiac group included children with a clinical diagnosis of MIS-C without cardiac involvement (n = 9), children with fever plus prior/current SARS-CoV-2 detected on RT-PCR (n = 75), and children with fever and serologic evidence of SARS-CoV-2 antibodies (n = 14). All cardiac MIS-C patients (15/15) had MDW values above 24.0 (100% sensitivity). Although MDW > 24.0 only carries 49% specificity, it is important to note that MDW was significantly increased in cardiac MIS-C compared to the non-cardiac patient group (mean 33.6 vs. 25.3, P < 0.0001, Fig. 4A). For these two groups of patients, we calculated an AUROC of 0.84 (Fig. 4B). This result highlights the utility of MDW for flagging individuals with potential cardiac involvement of MIS-C for additional investigations.

### Monocyte anisocytosis tracks clinical improvement

In children with MIS-C, MDW declined significantly during hospital course from a mean of 31.2 at admission and before treatment to a mean of 26.7 following treatment (Fig. 4C). MDW declined further to a mean of 18.2 at the time of discharge or follow-up, equivalent to MDW values of healthy control subjects and reflecting the expected resolution of illness (Fig. 4D). Interestingly, only two of the 16 MIS-C patients for which repeated blood samples were obtained displayed a peak value after admission to the hospital. These results suggest that MDW could be a helpful biomarker for tracking MIS-C disease progression, resolution under treatment, and the return to immune homeostasis.

### Monocyte anisocytosis outperforms other laboratory markers of MIS-C

**Fig. 5:**
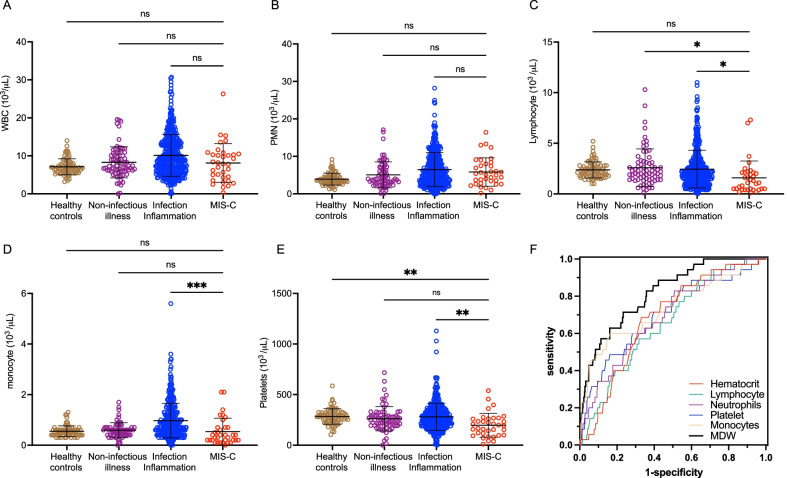
Assessment of other hematological parameters in MIS-C. Hematologic parameters, including **A** white blood cell (WBC), **B** neutrophil (PMN), **C** lymphocyte, **D** monocyte, and **E** platelet counts were compared between healthy controls, children with non-infectious illness, children with an infectious/inflammatory illness, and children with MIS-C in the validation cohort. Analysis by ordinary one-way ANOVA. ns = non-significant, * P < 0.05, ** P < 0.01, *** P < 0.001. **F** Receiver operator curve of each hematologic parameter in MIS-C compared to values obtained from children presenting for medical care for infection/inflammatory or non-infectious illness

We then sought to determine how detection of monocyte anisocytosis performed compared to other established, standard hematologic parameters to determine if quantifying monocyte anisocytosis added value to a standard CBC when screening for MIS-C. According to current guidelines, we focused on abnormalities in lymphocyte, neutrophil, and platelet counts, which are criteria for triggering additional MIS-C workup. [8] We found that total white blood cell count (WBC) could not distinguish between MIS-C, other infections, or healthy controls (Fig. 5A). We also found no differences in neutrophil count across any cohorts (Fig. 5B). Neutrophilia, a tier 1 biomarker [8], was present in 31.4% of the MIS-C patients (16 out of 51 for whom neutrophil counts were available), and we found no differences in neutrophil counts across any cohorts (P = 0.88, Fig. 5B). While total lymphocytes and monocytes counts were decreased in MIS-C compared to children with other infections, there were no differences when comparing these cell counts with healthy control subjects (Fig. 5C, D). The average neutrophil to lymphocyte ratio in MIS-C was 6.7 (SD 6.3), which was higher than the average 4.0 (SD 5.2) in children with infection/inflammatory illnesses (P < 0.001). The calculated AUROC for neutrophil to lymphocyte ratio in distinguishing MIS-C from infection/inflammation of other causes was 0.69.

Platelet counts were decreased in MIS-C (20 out of 51) compared to healthy control subjects and children with other infections (Fig. 5E, ANOVA, P < 0.01). However, a comparison of AUROC analyses of these CBC parameters reveals MDW as having the highest screening accuracy for MIS-C (Fig. 5F). Overall, current hematologic parameters have limited capability for distinguishing MIS-C from other illnesses, but monocyte anisocytosis, which can be detected as part of a CBC, offers a significant advantage over other hematologic parameters in identifying MIS-C (Fig. 5F).

We then sought to compare the detection of monocyte anisocytosis against CRP and ESR, two other clinical laboratory tests recommended in the tiered diagnostic evaluation of MIS-C [8]. Early and peak clinical laboratory values were compared between patients with MIS-C or patients with infectious and non-infectious controls. The subjects included in this analysis were the same as those in the validation cohort for which an MDW value was measured (n = 501). Early and peak CRP ≥ 5 mg/dL had 100% sensitivity but only 15.5 and 21.2% specificity, respectively, for identifying MIS-C patients. Early and peak ESR ≥ 40 mm/h had 72.2 and 78.8% sensitivity and 69.5 and 66.8% specificity, respectively, for MIS-C. These values reveal limitations in the specificity for CRP and sensitivity for ESR as biomarkers for MIS-C. Both tests peaked an average of 2.6 days into the hospital course, pointing towards their limitations as tier 1 assays for early identification of MIS-C. Even a combination of hematologic and inflammatory parameters from tier 1 testing (ESR > 40 or CRP > 5; platelets < 150,000 × 10^3^/µL or neutrophils > 6000 × 10^3^/µL) detect only 51.4% true positives with MIS-C, and this combination of parameters only displays 50% sensitivity and 50% specificity in distinguishing MIS-C from children with other infection/inflammatory illnesses.

## Discussion

We analyzed blood samples from a total of 762 children presenting for medical care and discovered prominent monocyte anisocytosis, detectable on routine CBC, in children with MIS-C. MDW, a hematologic parameter that quantifies monocyte anisocytosis, above a value of 24.0 serves as a useful threshold in tier 1 screening for MIS-C with a 100% sensitivity in identifying subjects with MIS-C with cardiovascular complications among children with fever in the setting of prior SARS-CoV-2 infection.

Currently, the American College of Rheumatology recommends a stepwise approach for laboratory and imaging workup for diagnosing MIS-C [8, 27], starting with Tier 1 testing, which includes a complete blood cell count (CBC), complete metabolic panel, C-reactive protein (CRP), and erythrocyte sedimentation rate (ESR). Abnormal results trigger a more comprehensive Tier 2 panel of MIS-C laboratory tests including ferritin, troponin, and N-terminal pro b-type natriuretic peptide (NT-proBNP), followed by cardiac evaluation and multidisciplinary subspecialist consultations. However, as we have shown, the initial screening tests are often abnormal in a range of infectious and non-infectious disease processes, limiting their ability to inform a diagnosis of MIS-C. Moreover, because children are often only mildly symptomatic or asymptomatic when acutely infected with SARS-CoV-2, a prior history of COVID-19 may not be established before a child presents with symptoms consistent with MIS-C. Therefore, our comprehensive assessment of how MDW could be used in screening for MIS-C reflects a practical approach for evaluating a screening tool for MIS-C.

In the relevant clinical context, an elevated MDW would urgently and efficiently prompt further evaluation of MIS-C, thereby serving as a much-needed tool to improve currently recommended MIS-C evaluation guidelines. We showed that MDW over 24.0 displays an 86% sensitivity in identifying children with MIS-C among patients presenting with general signs of infection. There was a slight decrease in sensitivity and specificity metrics in the validation cohort as compared to the discovery cohort, which may be explained by the evolving knowledge around MIS-C, leading to earlier recognition of MIS-C by clinicians and the diagnosis of a larger number of subjects with milder MIS-C. Other factors may include the altered MIS-C pathogenicity by SARS-CoV-2 variants and the broader range of severe viral and bacterial infections after the end of lockdowns.

Monocyte anisocytosis offers additional advantages over other inflammatory parameters used in identifying MIS-C: it can be detected as part of the routine CBC. Obtaining all recommended tests is time and resource-intensive, requires collection in blood tubes with different anticoagulants, and clinical practice varies significantly between medical centers. [28] Because monocyte anisocytosis can be reported on routine CBCs and MDW shows high sensitivity with high screening accuracy for MIS-C, obtaining MDW with the initial laboratory assessment could help streamline the evaluation process, reduce the volume of blood collected from pediatric patients, and expedite diagnostic evaluations and therapeutic interventions if indicated. Importantly, monocyte anisocytosis is most prominent upon presentation, making it a useful early screening tool, and decreases with treatment, and several weeks after completion of MIS-C treatment, MDW values return to the range of values observed in healthy subjects.

It is important to note that monocyte anisocytosis is not specific to MIS-C and can be seen in other acute illnesses. In-depth immune profiling of children with MIS-C also revealed increased markers of monocyte activation, such as increased CD64 expression [29], increased ICAM1 expression [29], and decreased CD16 expression [15, 30]. Similar changes in monocytes, reflected in MDW above a threshold of 20, have been reported in sepsis and organ dysfunction in children [16, 17] and adults [18–20], trauma [31], and viral infections like COVID-19 [32, 33], and influenza [34]. Thus, monocyte anisocytosis should be used as a MIS-C screening test in concert with clinical findings as it is not a stand-alone diagnostic test. Additionally, not all hematology analyzers today offer the option of measuring MDW, however, as current and further studies help clarify the utility of MDW in acute illness, it is likely that this marker of monocyte activation will become a more accessible option on all hematology analyzers.

## Conclusion

While the number of COVID-19 cases continues to rise, children continue to be at risk of MIS-C, a delayed-onset, potentially life-threatening hyperinflammatory syndrome [35]. Monocyte anisocytosis reflects the monocyte-mediated hyperinflammation driving MIS-C, and MDW, which can be obtainable as part of a CBC early in the clinical workup, improves the identification of children with cardiac involvement during MIS-C, thereby improving current practice guidelines.

## Data Availability

The datasets generated and/or analyzed during the current study are not publicly available due MassGeneral Brigham policies. However, data used in this study may become available to other researchers upon reasonable request to the corresponding author and in compliance with the MassGeneral Brigham Innovations Office.

## Abbreviations

AUROC: Area under the receiver operator curve
CBC: Complete blood count
CDC: Centers for Disease Control and Prevention
CRP: C-reactive protein
ED: Emergency Department
ESR: Erythrocyte sedimentation rate
FDA: Food and Drug Administration
IRB: Institutional Review Board
K2 EDTA: Di-potassium ethylenediaminetetraacetic acid
MDW: Monocyte distribution width
MIS-C: Multisystem inflammatory syndrome in children
NT-proBNP: N-terminal pro b-type natriuretic peptide
