# Correction to: Monocyte anisocytosis increases during multisystem inflammatory syndrome in children with cardiovascular complications

**DOI:** 10.1186/s12879-022-07563-4

**Published:** 2022-07-07

**Authors:** Lael M. Yonker, Oluwakemi Badaki-Makun, Puneeta Arya, Brittany P. Boribong, Gabriela Moraru, Brittany Fenner, Jaimar Rincon, Alex Hopke, Brent Rogers, Jeremiah Hinson, Alessio Fasano, Lilly Lee, Sarah M. Kehoe, Shawn D. Larson, Hector Chavez, Scott Levin, Lyle L. Moldawer, Daniel Irimia

**Affiliations:** 1 Department of Pediatrics, Massachusetts General Hospital, 55 Fruit Street, Boston, MA 02114 USA; 2 Mucosal Immunology and Biology Research Center, Massachusetts General Hospital, 55 Fruit Street, Boston, MA 02114 USA; 3 Harvard Medical School, Boston, MA USA; 4 Department of Pediatrics, Johns Hopkins School of Medicine, Baltimore, MD USA; 5 Center for Data Science in Emergency Medicine, Johns Hopkins University, Baltimore, MD USA; 6 Jackson Memorial Hospital, Miami, FL USA; 7 Holtz Children’s Hospital, Miami, FL USA; 8 Department of Surgery, University of Florida, Gainesville, FL USA; 9 Danaher Diagnostics LLC, Boston, MA USA; 10 Department of Emergency Medicine, Johns Hopkins School of Medicine, Baltimore, MD USA; 11 Department of Surgery, Center for Engineering in Medicine, Massachusetts General Hospital, 114 16th Street, Boston, MA 02129 USA; 12 Shriners Burn Hospital, Boston, MA USA

## Correction to: BMC Infectious Diseases (2022) 22:563 https://doi.org/10.1186/s12879-022-07526-9

Following the publication of the original article [1], the authors identified that some symbols were absent in the on-line version of Figs. 4 and 5. These Figures have been corrected.

**Fig. 4:**
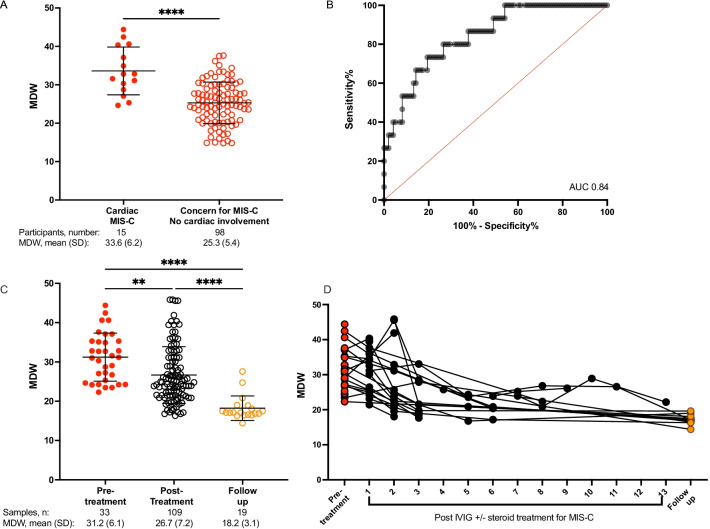
MDW depends on MIS-C severity and changes through the course of MIS-C diagnosis, treatment, and recovery. **A** Higher MDW values in MIS-C patients who manifested cardiac complications (Cardiac MIS-C) compared to children with MIS-C without cardiac involvement or presenting with symptoms concerning MIS-C (fever plus recent/current positive SARS-CoV2 PCR or SARS-CoV2 antibodies positive). **B** ROC in the validation cohort to assess the utility of MDW as a screening tool for cardiac involvement of MIS-C. AUC = area under the curve (fraction). **C** Blood from children with MIS-C was collected at multiple time points. MDW was plotted by time of collection: at admission, during hospital course, and at discharge or follow-up. Analysis by one way ANOVA. **P < 0.01, ****P < 0.0001. **D** MDW values from individual patients with MIS-C are plotted over the course of their illness. Black lines connect individual patients with MIS-C. Not all patients provided blood samples at each time point

**Fig. 5:**
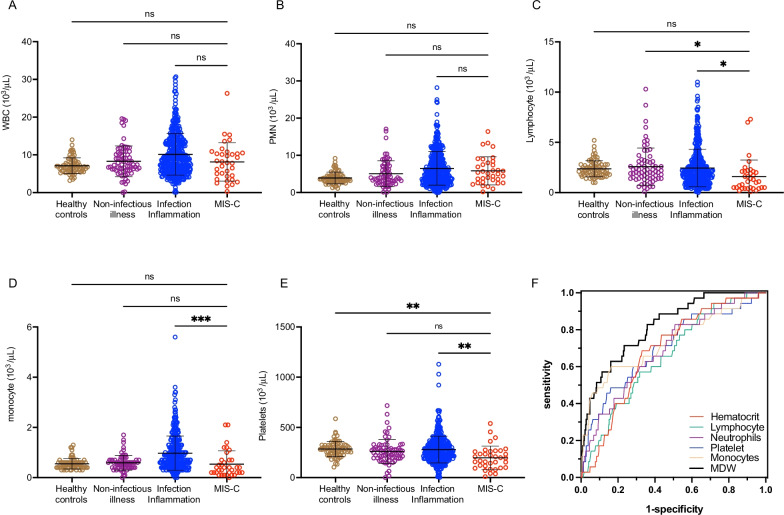
Assessment of other hematological parameters in MIS-C. Hematologic parameters, including **A** white blood cell (WBC), **B** neutrophil (PMN), **C** lymphocyte, **D** monocyte, and **E** platelet counts were compared between healthy controls, children with non-infectious illness, children with an infectious/inflammatory illness, and children with MIS-C in the validation cohort. Analysis by ordinary one-way ANOVA. ns = non-significant, * P < 0.05, ** P < 0.01, *** P < 0.001. **F** Receiver operator curve of each hematologic parameter in MIS-C compared to values obtained from children presenting for medical care for infection/inflammatory or non-infectious illness

The original article has been corrected.
